# Suppressing Health-Related Thoughts: A Pathway to Increased Anxiety?

**DOI:** 10.1192/j.eurpsy.2025.1014

**Published:** 2025-08-26

**Authors:** N. Boussaid, R. Feki, I. Gassara, H. E. Mhiri, S. Omri, N. Charfi, J. Ben Thabet, M. Maalej, N. Smaoui, M. Maalej, L. Zouari

**Affiliations:** 1 Psychiatry C, Hedi Chaker university hospital center, Sfax, Tunisia

## Abstract

**Introduction:**

Health-related anxiety, characterized by excessive worry about one’s health, often leads to significant distress and impairment. Thought suppression, the conscious attempt to control or avoid unwanted thoughts, is frequently associated with various psychological conditions, including anxiety disorders. Research suggests that individuals with anxiety may attempt to suppress thoughts related to their fears, potentially exacerbating symptoms.

**Objectives:**

The present study investigates the relationship between thought suppression and health-related anxiety, exploring how the tendency to suppress intrusive thoughts may influence health anxiety levels.

**Methods:**

A cross-sectional survey design was employed, with participants drawn from a sample of medical students. The White Bear Suppression Inventory (WBSI) was used to assess the extent to which participants tend to suppress intrusive thoughts, while the Health Anxiety Inventory (HAI-18) was used to measure health-related anxiety. The HAI-18 evaluates the frequency and intensity of health-related worries and behaviors over the past six months.

**Results:**

The study recruited 213 medical students, of which 74.2% were female. The mean age of participants was 22.11 ± 2 years. Among the sample, 22.1% had a personal medical history, and 20.2% had a documented history of psychiatric disorders. Regarding family medical history, 59.6% of participants reported a familial history of medical conditions, and 21.6% reported a familial history of psychiatric disorders. Additionally, 39% of participants had family members who had been hospitalized for serious illness.

Health-related anxiety was observed in 26.3% of participants. A significant positive correlation was found between WBSI scores (thought suppression) and HAI scores (health-related anxiety) (r = 0.301, p < 0.001), suggesting that participants who reported higher levels of thought suppression were more likely to experience health-related intrusive thoughts and heightened anxiety about their health. Furthermore, individuals with higher health anxiety tended to have a stronger inclination to suppress thoughts, compared to those with lower levels of health anxiety.

**Conclusions:**

The findings indicate a positive relationship between thought suppression and health-related anxiety among medical students. Specifically, students who engage in higher levels of thought suppression tend to report more frequent intrusive thoughts related to health and greater anxiety about their well-being. Future research could explore interventions aimed at reducing thought suppression as a potential strategy for alleviating health anxiety.

**Disclosure of Interest:**

None Declared

